# Oversized Planer Shavings for the Core Layer of Lightweight Particleboard

**DOI:** 10.3390/polym13071125

**Published:** 2021-04-02

**Authors:** Jakob Gößwald, Marius Cătălin Barbu, Alexander Petutschnigg, Ľuboš Krišťák, Eugenia Mariana Tudor

**Affiliations:** 1 Forest Products Technology and Timber Construction Department, Salzburg University of Applied Sciences, Markt 136a, 5431 Kuchl, Austria; jakob.goesswald@fh-salzburg.ac.at (J.G.); cmbarbu@unitbv.ro (M.C.B.); alexander.petutschnigg@fh-salzburg.ac.at (A.P.); 2 Faculty of Furniture Design and Wood Engineering, Transilvania University of Brasov, B-dul. Eroilor nr. 29, 500036 Brasov, Romania; 3 Paris Salzburg Center for Smart Materials, c/o Department of Chemistry and Physics of Materials, Lodron University of Salzburg, Jakob-Harringer-Strasse 2A, 5020 Salzburg, Austria; 4 Institute of Wood Technology and Renewable Materials, University of Natural Resources and Life Sciences (BOKU), Konrad Lorenz-Straße 24, 3340 Tulln, Austria; 5 Faculty of Wood Sciences and Technology, Technical University in Zvolen, T.G. Masaryka 24, 96001 Zvolen, Slovakia; kristak@tuzvo.sk

**Keywords:** planer shavings, particleboard, plowshare mixer, drum mixer, pigment

## Abstract

Planer shavings (PS) are side-products generated during the processing of solid wood, typically used for heating, packaging, or insulation purposes. PS has been used for decades in particleboard manufacture, particularly in the core layer. The aim of this research is to investigate the use of PS with a length over 4 mm in low-density one-layer particleboard manufacturing with a thickness of 10 mm, as an option to reduce the raw material demand for wood-based panels. Correlations towards the mechanical properties of the particleboards, fabricated at a density of 475 kg/m^3^, could be drawn by analyzing the effects of different urea-formaldehyde adhesive contents (6%, 9%, and 12%). Two methods of adhesive application (pouring and spraying) and two types of blending of PS with adhesive (plowshare mixer and drum mixer) were investigated, with the aim that PS will have controlled resin application. The difference between the adhesive application methods was examined by analyzing the mechanical properties as an internal bond, modulus of rupture, and modulus of elasticity as well as indirectly by visualizing the adhesive distribution by adding a green pigment to the adhesive before application. PS demonstrated reduced bending properties in comparison with the EN 312 standard requirements of particleboards for internal use in dry conditions (type P2), due to the low density. The internal bond strength in the case of the particleboard without pigment application (up to 0.5 N/mm^2^) was higher compared to the P2 requirements (0.4 N/mm^2^), and significantly lower (0.15 N/mm^2^) in combination with the pigment (2.5% based on the board weight, compared to 0.1%, specific for such industry applications), but still superior to the values of the reference panel manufactured with wood particles.

## 1. Introduction

Nearly all types of woodworking processes generate significant amounts of by-products and residues [1]. This material is used for a determined period and then subsequently recycled as wood shavings [2], disposed of as residues from primary wood processing, e.g., sawdust, trimming residues, wood pieces, chips and bark [3,4], or combined with other materials to form composites [5]. These by-products and leftovers result from the processing of the logs and, added to the branches, trunks, and roots that remain in the forest, represent a significantly higher volume than those of the wood product obtained [6]. These wood processing residues are generated at industrial facilities and are easily collectible and reusable as potential feedstocks for the wood-based panel, pulp, and pellet industry.

The intrinsic physical properties combined with their availability and low cost endorse the utilization of wood shavings for multiple purposes [7]. The environmental impacts can be diminished by using wood by-products in particular [2,4]. Abu-Jdayil et al. (2019) [8] analyzed the renewable thermal building insulation materials including wood shavings. The thermal properties of phase-changing materials (able to release, absorb or store sufficient energy at phase transition to provide useful heat/cooling) combined with wood shavings were studied by [5]. The thermal insulation performance of concrete was improved by adding wood shavings while decreasing its compressive strength [9,10,11,12]. The waste materials reduce the heat transfer considerably, especially in a dry state [10]. The addition of wood shavings and straw in the composition of lightweight sand concrete resulted in improved physical and mechanical properties [11]. Wood shavings replacing fine aggregates combined with mortar were studied by [12,13]. When used in the livestock sector, as bedding material [14], the wood waste has to be free of any contaminants [15]. Wood-waste management practices and quality requirements for the use of wood residues into value added products, including here planer shavings, were analyzed by [16].

Harkin (1969) [7] described three utilizations of fuel uses for wood shavings: (a) for power and heat at the production plant, combined with other wood waste; (b) in public buildings and power plants; (c) as briquettes. A further utilization for thermal purposes is in pellet production [17]. Both sawdust and wood shavings are useful as a base for practice ski slopes in dry areas [18]. Other utilizations are in pulp production [19,20], packaging [7], fish smoking [21], abrasive materials (hand soaps, metal polishes), particleboards [22] as well as wood fiber-based composites, and floor coverings [7].

The production of particleboard comprising residues and by-products such as sawdust, planer shavings, and other residues, started about 80 years ago in the USA and Germany [1,23]. These residues were included mostly in the core layer of these boards [6,24].

The wood-based panel industry is facing increased competition for wooden raw materials from the renewable energy sector, due to the current legislative requirements promoting the use of wood for producing bioenergy to meet the determined renewable energy targets. The use of wood side products for the manufacture of particleboard can reduce environmental impacts and ensure an upscaling for this raw material for the production of wood-based composites [25]. Studies that included wood waste or residues for the production of particleboard have been carried out for more than three decades. Researchers investigated the properties of particleboards made from decayed wood and bark [26], pine needle litter [27], sunflower stalks [28], sunflower seed husks [29], almond shells [30], walnut and hazelnut shells [31], brewer’s spent grain [32], pruned branches [33] and wood chip wastes [34,35]. Rowell (2014) [6,24] analyzed the utilization of wood shavings waste (*Populus euroamericana* and *Eucalyptus grandis*) for the production of particleboards [1]. Another way to lower negative environmental impacts is the use of wood waste-based adhesives as particleboard panel binders [36,37,38,39,40].

The objective of this study was to assess the mechanical and physical properties of particleboard made with a mixture of oversized planer shavings from spruce, pine, and beech, the common wood species processed in workshops in Central Europe by determining the internal bond, modulus of rupture, modulus of elasticity, thickness swelling and water absorption of the panels. In this research we evaluated the effects of adhesive application by spraying and pouring, the influence on the panel’s properties of two mixing systems for wood particles and adhesive, namely plowshare and drum mixer.

## 2. Materials and Methods

The PS was obtained from the workshops of Holztechnikum Kuchl, where these residues were collected after the planning of dry sawnwood. The particle mixture of spruce (*Picea abies*), pine (*Pinus sylvestris*), and beech (*Fagus sylvatica*) was selected with the fraction over 4 mm, containing flat to curved shavings, which were used as raw material. At normative climate (20 °C and 65% relative air humidity) the moisture content of these was 10.5%.

The target density of all boards was set to 475 kg/m^3^, close to the raw density of spruce, to avoid big voids inside the board, but to still avoid the over densification of the particles. The reference board was manufactured using spruce (*Picea abies*) particles for the core layer, supplied by Kaindl Company (Wals, Austria). These coarse-grained wood particles had an elongated, double pyramid-like shape, at a density of 475 kg/m^3^. Urea-formaldehyde (UF) type 10F102 adhesive, with a solid content of 66%, was sourced from MetaDynea Company (Krems, Austria). One percent ammonium sulfate was used as a hardener. The raw material (PS and core wood particles) was blended with 6%, 9%, and 12% UF resin for 5 min in a plowshare mixer ENT type WAM WHB-75 (Laarne, Belgium) and drum mixer ATIKA BM 125 S (Burgau, Germany). In the plowshare mixer, the materials are projected in a three-dimensional manner on the inside wall of the drum, giving the possibility to merge with each other. The drum mixer operates with a flanged opening for easy filling and metal wheels on the chassis. Spraying the resin with the spray gun Metabo FSP 600 (Nürtingen, Germany), within 60 sec was the first application method tested, while at the second method (pouring), the resin was slowly discharged directly out of the measuring tank into the mixer. The 400 × 400 mm one-layer particleboards, with a thickness of 10 mm and density of 475 kg/m^3^ were pressed with a hydraulic press, Höfer HLOP 280 (Taiskirchen, Austria), at 180 °C with a press time factor of 33 s/mm (pressing time 5.5 min), with three replications for each board. To visualize the effectiveness of mixing type, 19 g of green pigment of the company Habich (Leiben, Austria) was added in the formulation of each panel (Table 1).

The samples were codified as follows. For pigment (color): C, for adhesive application type; P, for pouring; and S for spraying. For mixer type: M, for plowshare mixer; and D, for drum mixer (Table 1). Adhesive amount (%) was also indicated in the codification of samples. The reference panel with spruce particles had a resination factor of 12% adhesive and 2.5% pigment.

The following tests were performed: internal bond EN 319:2005 [41], 3-point bending strength and modulus of elasticity EN 310:2005 [42], thickness swelling, and water absorption after 24 h EN 317:2005 [43]. The testing specimens were cut according to EN 326-1:2005 [44]. The mechanical tests were carried out on the universal testing machine Zwick Röll Z 250 (Ulm, Germany). The density profile used as a further board property indicator was performed on the Dense-Lab X (EWS, Hameln, Germany).

## 3. Results and Discussion

### 3.1. Raw Material Characterization

The wood shavings (PS) are shown at a sample size of 50, a loosening factor of 17.5, and an average particle size of 7.8 mm in the fiber direction, 10.3 mm in width and 0.23 mm in thickness, and an average degree of slenderness of 27. This resulted in a very high volume of the particles in comparison to the geometry of reference wood particles (WP) for the core.

**Figure 1:**
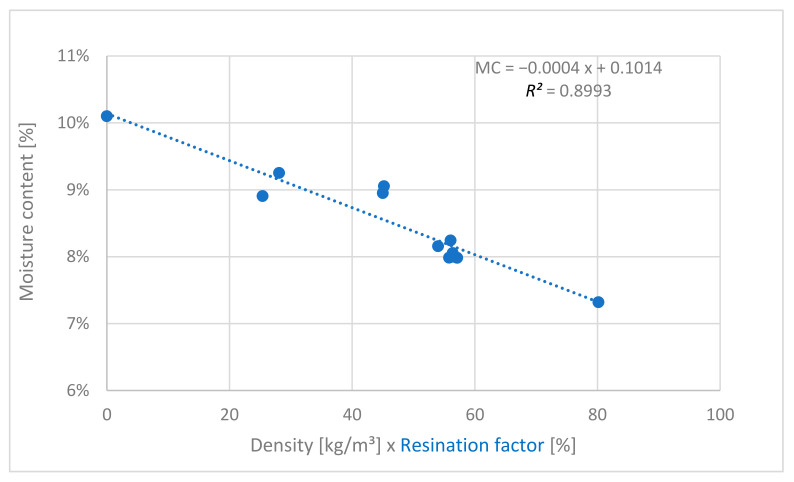
Correlation between moisture content (MC) vs. density and resination factor.

The moisture content (MC) was evaluated according to EN 322:2005 [45] for the produced boards with a very low coefficient of variance, under 3%, for all panels (after climatization at 20 °C and 65% relative air humidity). Because the MC could be influenced by the density and the resination factor, regression analysis was performed, by multiplying the two variables (Figure 1). Also, this model was verified using an *F*-test, to be the best suitable combination of the variables, as the highly significant empirical *F* value of 89 and the *R*^2^ value of 0.9 (Figure 1) indicates.

### 3.2. Thickness Swelling and Water Absorption

**Figure 2:**
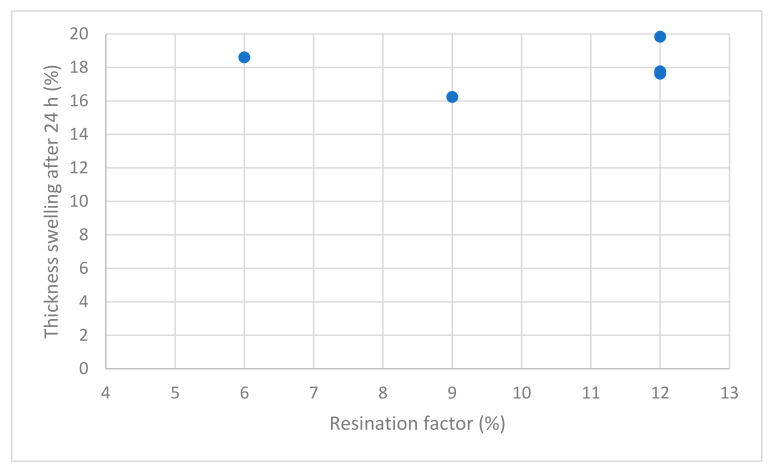
Correlation between thickness swelling (TS) after 24 h, and resination factor (6%, 9%, and 12% UF).

Thickness swelling (TS) after 24 h (Table 2), measured according to EN 317:1993 [43], of all particleboard manufactured with PS was significantly lower compared to the reference. A correlation between resination factor and TS cannot be defined, with an *R*^2^ of 0.01 (Figure 2). The PS samples with 6% glue with UF absorbed more water compared to the testing specimens bonded with 9% and 12% UF, respectively.

Water absorption (WA) after 24 h was measured according to EN 317:1993 [43]. PS showed a 10% reduction in WA (from 47% to 54%) compared to reference boards manufactured with wood particles (60%). The samples with 9% resin content showed significantly lower WA, while the samples with an adhesive content of 6% and 12% showed higher values and no significant difference between each other. Thus, absolutely no linear correlation between WA and resination factor could be determined.

Particleboards manufactured with PS were characterized by higher contact between particles, which resulted in the improvement of their glue bonds and reduced TS values. Similar to the case of WA, high contact particles represent a physical barrier to water intake, resulting in a decreased void among particles [46].

### 3.3. Mechanical Properties

The average values of the mechanical properties internal bond (IB), modulus of rupture (MOR), and modulus of elasticity (MOE) are presented in Table 3.

#### 3.3.1. Internal Bond

**Figure 3:**
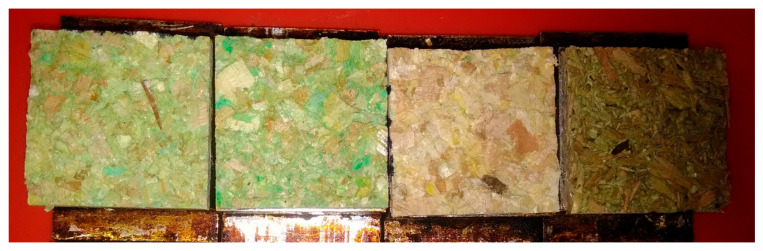
Green discoloration by added pigments on broken IB samples from left to right: C12S_M, C12P_M, C12S_D, and reference.

The analysis of variance was performed with an empirical *F* value of 17.7 at a significance level of 0.05. Despite a relatively high coefficient of variation (17–27%), the influence of the resin application method is highly significant. By comparing the sprayed samples, C12S_M (IB = 0.15 N/mm^2^), with the poured ones, C12P_M (IB = 0.10 N/mm^2^), a loss of strength can be identified at the IB values as well as an increased number of green spots (Figure 3). Also, the color of the board changes to a lighter green, when the adhesive is sprayed. This indicates that the IB benefits from the homogeneous distribution of the resin and emphasizes the use of pigments as a way of visualizing the adhesive distribution when mixed with the raw material. On the other hand, the C12S_D samples showed almost no coloration. It is assumed that a large amount of adhesive was lost out of the mixer during application because the drum mixer couldn’t be tightened. Therefore, the gluing method with the drum mixer turned out to be unsuitable for this purpose. The reference specimens showed a homogenous resin distribution but at a level of IB of 0.06 N/mm^2^, which did not reach the measured values of C12S_M.

The average IB values showed significantly higher values for the specimens 6S_M and 9S_M with a lower resination factor and without pigments mixed with the adhesive when 2.5% pigment was mixed with the adhesive, IB decreased considerably at 0.06 N/mm^2^ for the reference and between 0.04 and 0.15 N/mm^2^ for the samples with 12% resination. The pigment seems to decrease the IB of the boards and explains the unusual negative correlation of the adhesive content with the IB. Also, the low *R*^2^ value of 0.35 indicates a poor correlation, even if the strong variance within the series is considered. The samples without pigments were demonstrated to be able to meet the standard requirement for P2 particleboard (0.4 N/mm^2^) at the resination factor of 9%.

#### 3.3.2. Modulus of Rupture and Modulus of Elasticity

**Figure 4:**
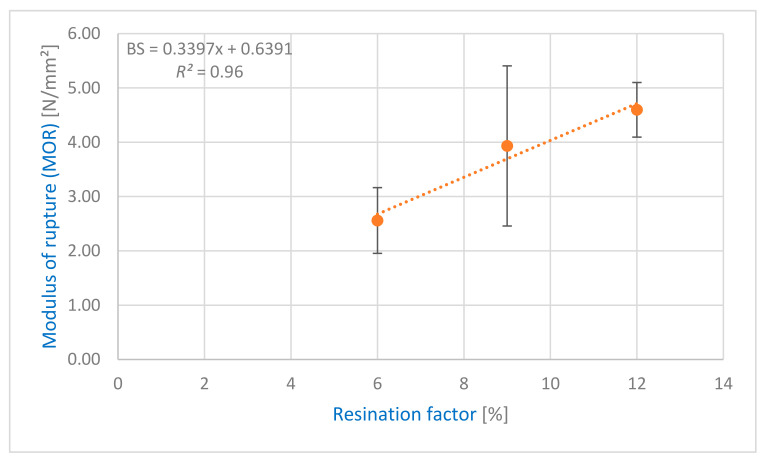
Regression model of resination factor (6%, 9%, and 12% UF), and modulus of rupture (MOR) of the light particleboard with PS.

**Figure 5:**
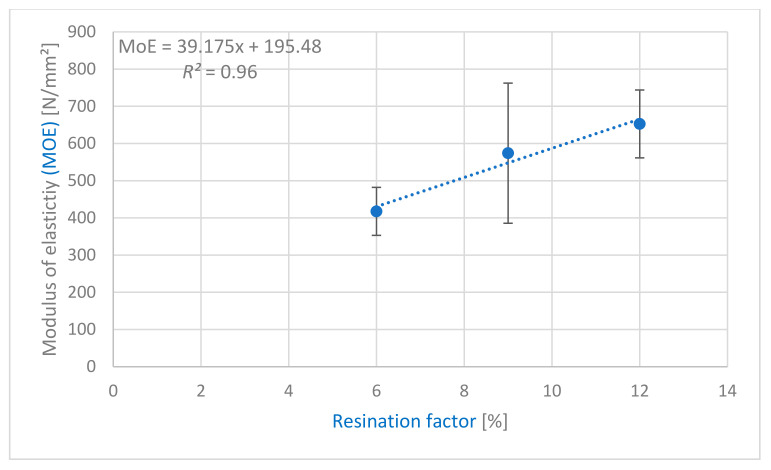
Regression model of resination factor (6%, 9%, and 12% UF), and modulus of elasticity (MOE) of the light particleboard with PS.

Similar to the IB, analysis of variances was performed with an empirical *F* value of 6.5 at a significance value of 0.05 for the MOR values. The lower empirical *F* value indicates that the MOR is less influenced by the adhesive application method than the IB. Especially samples C12S_M, with a MOR of 4.60 N/mm^2^, and C12P_M (4.5 N/mm^2^) showed an insignificant difference. However, the process with the sprayed adhesive application (C12S_M) seems to be more stable compared to the poured ones, because the variance of the samples showed a 5.7% coefficient of variation and the results showed a less inclined distribution. Samples C12S_D were on the contrary, with a MOR of 2.6 N/mm^2^ underlining the unsuitability of the used drum mixer, as outlined in 3.3.1 (IB). Figure 4 shows a high positive correlation (*R*^2^ = 0.96) of the adhesive content (%) with the MOR. The positive correlation does not show any significant negative impacts, that could have been caused by the pigment, and its influence on the MOR and MOE is neglectable (Figure 4 and Figure 5).

Based on previous research [47], coarse wood shavings resulted in a higher modulus of rupture.

The remaining differences can be explained by an almost 10% change in the density and different resination factors in 9S_M and 6S_M [48,49].

The reference boards showed an average MOR of 2.7 N/mm^2^ and a median of 1.9 N/mm^2^ indicating a high variance inside the board, as demonstrated in Table 1. The lower MOR values in comparison to C12S_M were caused by the used material, which had a lower average particle size and an elongated shape similar to a double pyramid. MOE of all tested specimens was under 700 N/mm^2^. The lowest MOE was measured for the sample whose particles were blended with resin in a drum mixer. With the exception of 6S_M, all other samples showed higher MOE compared to the reference.

Samples C12S_M had both an MOR of 4.6 N/mm^2^ and a MOE of 653 N/mm^2^, as higher values compared to the rest of the samples. None of the tested boards were nearly able to meet the requirements of the P2 standard, on the one hand, because only a low-density board made only of core layer was produced, and on the other hand, because of the density profile (no poignant face layers).

### 3.4. Density Profile

**Figure 6:**
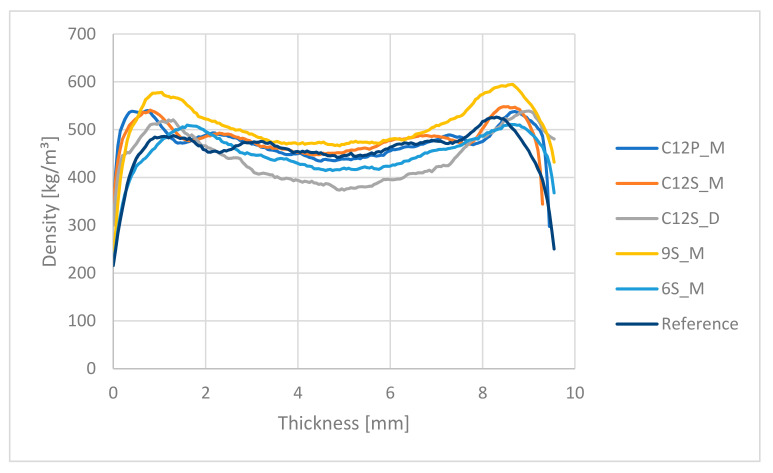
Density profile of the one-layer panels with PS and wood particles.

The peak densities of the edges are in many cases over 1 mm inside of the board, especially at those boards, which show a lower MOR. Such density profiles are not typical for industrial particleboard, due to the pressing in laboratory conditions. Moreover, these panels were not sanded prior to testing. The density profile is not distinct compared to the three-layered particleboard, since the difference between the peaks (which represent the faces of the panel) and the middle of the core (which represents the middle of the panel) for most boards is less than 70 kg/m^3^ (Figure 6). The minimum core density of less than 400 kg/m^3^ and a face-core density difference of 120 kg/m^3^ was achieved by the boards which were blended in the drum mixer (C12S_D). The highest density of the peaks (600 kg/m^3^) was measured for the boards 9S_M, blended in a plowshare mixer. The explanation for the density profile for all manufactured panels can be attributed to the manual scattering of the planer shavings in the mold.

In the case of an industrially manufactured 10 mm P2 particleboard with an average density of over 700 kg/m^3^, the peaks reach a density over 800 kg/m^3^ and a core density of about 500 kg/m^3^, which can easily explain why the MOR, and partially IB, are superior to the laboratory manufactured PS panels.

## 4. Conclusions

The results of this study have revealed that particleboards of a low density, made of PS, are able to achieve IB values over the requirement of the P2 standard at a UF adhesive content of 9% (without pigment). Due to the lower density (about 2/3 of a standard 10 mm P2 type) single-layer structure and a relatively plain density profile, lower MOR (4 N/mm^2^) and MOE (600 N/mm^2^) were measured.

The highest mechanical properties of the adhesive application test series were obtained by spraying adhesive inside a plowshare mixer, other application methods, like pouring, result in unacceptable performance and increases the variation of the resulting low-density boards. The homogeneity of the adhesive distribution can be easily visualized by adding a pigment to the glue, however with the drawback of significantly reducing the IB in comparison to none treated boards. Other board properties, on the other hand, do not seem to be influenced noticeably. The use of a plowshare mixer had a result in a better blending of planer shavings and wood particles compared to a drum mixer.

Because of the smaller particle size of the reference boards, their performance at the same degree of gluing, was inferior to PS, even if the stiffness was revealed to be at the same MOR, or higher, for the reference samples. Furthermore, it could be statistically proven that the MC of the final boards can be better predicted by the product of density and adhesive content.

The increased percentage of pigment (2.5%) compared to the usual 0.1% applied in industrial production negatively influenced all the physical and mechanical properties of the manufactured PB. However, further efforts need to be taken in this case to overcome the issues of low MOR and MOE, for example by applying a three-layered structure and modifying the pressing cycle to obtain higher densities in the surface layers.

## Figures and Tables

**Table 1:** Experimental design for the particleboard fabricated with wood shavings bonded with 6%, 9%, and 12% urea-formaldehyde (UF) resin.

Particleboard	Material Type	Density (kg/m^3^)	Thickness (mm)	Resination Factor (%)	Adhesive Application	Mixer Type
C12P_M	PS	475	10	12	pouring	Ploughshare
C12S_M	PS	475	10	12	spraying	Ploughshare
C12S_D	PS	475	10	12	spraying	Drum
9S_M	PS	475	10	9	spraying	Ploughshare
6S_M	PS	475	10	6	spraying	Ploughshare
Reference	WP *	475	10	12	spraying	Ploughshare

* WP = wood particles.

**Table 2:** Thickness swelling and water absorption after 24 h of the 10 mm particleboards with PS (values with the same letter (a, b, c, d, e) are not significantly different ANOVA, Post-Hoc Tukey HSD, *p* = 0.05; standard deviation in parentheses).

Particleboard Type	Thickness Swelling	Water Absorption	MC	Density
	(%)	(%)	[%]	(kg/m^3^)
C12P_M	17 ^a,b,c,d^ (1.8)	51 ^a,b^ (5)	8.1 ^a^ (0.1)	472 ^c^ (19)
C12S_M	17 ^a,b,c,d^ (2.5)	53 ^c^ (5)	8.0 ^a^ (0.1)	475 ^c^ (19)
C12S_D	18 ^a,b,c,d^ (2)	55 ^c^ (4)	8.0 ^a^ (0.1)	442 ^a^ (25)
9S_M	16 ^a,b,c,d^ (3)	47 ^a^ (6)	9.0 ^a^ (0.1)	501 ^d^ (17)
6S_M	19 ^a,b,c,d^ (2)	54 ^c^ (4)	9.1 ^a^ (0.2)	446 ^a^ (30)
Reference	26 ^e^ (2.6)	60 ^d^ (7)	8.0 ^a^ (0.1)	455 ^b^ (17)

**Table 3:** Internal bond, modulus of rupture, and modulus of elasticity of the 10 mm particleboards with PS (values with the same letter (a,b,c,d,e) are not significantly different ANOVA, Post-Hoc Tukey HSD, *p* = 0.05; standard deviation in parentheses).

Particleboard Type	IB	MOR	MOE
	(N/mm^2^)	(N/mm^2^)	(N/mm^2^)
C12P_M	0.10 ^b^ (0.02)	4.47 ^d^ (0.74)	593 ^c,d^ (134)
C12S_M	0.15 ^c^ (0.04)	4.60 ^d^ (0.50)	653 ^e^ (91)
C12S_D	0.04 ^a^ (0.01)	2.63 ^a,b^ (0.40)	259 ^a^ (50)
9S_M	0.48 ^e^ (0.16)	3.93 ^c^ (1.47)	574 ^c^ (188)
6S_M	0.34 ^d^ (0.15)	2.56 ^a,b^ (0.61)	418 ^b^ (65)
Reference	0.06 ^a^ (0.02)	2.69 ^a,b^ (1.34)	546 ^c^ (208)

## Data Availability

Not applicable.
